# Prognostic Value of Temporalis Muscle Thickness for Predicting Mortality in Patients with Emergency Department Cardiac Arrest

**DOI:** 10.3390/medicina62091760

**Published:** 2026-09-12

**Authors:** Ahmet Öztürk, Serkan Günay, Erdal Komut, Hakan Özerol, Mert Barındık, Serdal Ateş, Seval Komut, Yavuz Yiğit

**Affiliations:** 1 Department of Emergency Medicine, Hitit University Çorum Erol Olçok Education and Research Hospital, 19000 Çorum, Türkiye; drsrkngny@gmail.com (S.G.); drsevalkomut@hotmail.com (S.K.); 2 Department of Radiology, Hitit University Çorum Erol Olçok Education and Research Hospital, 19000 Çorum, Türkiye; erdalkomut@hotmail.com; 3 Department of Emergency Medicine, Gaziantep City Hospital, 27000 Gaziantep, Türkiye; ozerolhakanva@gmail.com; 4 Department of Emergency Medicine, Kocaeli City Hospital, 41000 Kocaeli, Türkiye; mertbarindik@gmail.com; 5 Intensive Care Unit, Dr. Abdurrahman Yurtaslan Ankara Oncology Training and Research Hospital, University of Health Sciences, 06000 Ankara, Türkiye; serdalmd@gmail.com; 6 College of Medicine, QU Health, Qatar University, Doha P.O. Box 2713, Qatar; yyigit@qu.edu.qa

**Keywords:** temporalis muscle thickness, emergency department cardiac arrest, cardiac arrest, resuscitation, mortality

## Abstract

*Background and Objectives*: Temporalis muscle thickness (TMT) has emerged as an imaging-based morphometric surrogate associated with reduced muscle mass and frailty; however, its prognostic value in emergency department cardiac arrest (EDCA) remains unclear. This study aimed to evaluate the association between TMT and emergency department mortality in patients with EDCA who had cranial CT imaging available during the same emergency department encounter and to investigate its association with in-hospital mortality. *Materials and Methods*: This study included 129 adult patients who experienced cardiac arrest in the emergency department between 1 June 2022 and 31 May 2025, and had eligible cranial CT imaging available during the same emergency department encounter. TMT was measured on non-contrast brain computed tomography images. The association between TMT and mortality outcomes, including emergency department and in-hospital mortality, was statistically analyzed. *Results*: TMT was significantly lower in patients who died in the emergency department compared with survivors (median 3.29 mm [Q1–Q3: 2.63–3.95] vs. 4.69 mm [Q1–Q3: 3.70–5.65], *p* < 0.001). Similarly, patients who died in an in-hospital setting had lower TMT values than survivors (median 4.06 mm [Q1–Q3: 3.26–5.34] vs. 5.00 mm [Q1–Q3: 4.21–5.85], *p* = 0.040). ROC analysis demonstrated the moderate discriminative ability of TMT for predicting emergency department mortality (AUC = 0.763, 95% CI 0.671–0.856). Logistic regression analysis identified that, after adjustment for age, sex, and CPR duration, TMT remained associated with emergency department mortality (adjusted OR 0.34, 95% CI 0.133–0.886; *p* = 0.027). *Conclusions*: Among patients with EDCA who had eligible cranial CT imaging during the same emergency department encounter, lower temporalis muscle thickness was associated with increased emergency department mortality, whereas its association with in-hospital mortality was weaker. Given the retrospective design and limited number of outcome events, these findings should be considered exploratory and require validation in larger independent cohorts.

## 1. Introduction

Cardiac arrest is defined as the sudden cessation of cardiac activity resulting in the loss of normal respiration or circulatory function. According to the location where it occurs, cardiac arrest is classified into two groups: in-hospital cardiac arrest (IHCA) and out-of-hospital cardiac arrest (OHCA) [1]. OHCA accounts for 73 emergency department visits per 100,000 people in the United States, whereas this rate is reported as 54 per 100,000 people in Europe [2]. Global estimates further indicate that IHCA occurs in 1–5 per 1000 hospitalized patients [3]. Emergency department cardiac arrest (EDCA) may constitute up to 19% of IHCAs, and due to its distinct characteristics, it has been suggested that EDCA may be classified as a third category of cardiac arrest [1,4,5]. Several studies have demonstrated that emergency department mortality rates among patients with EDCA range from 53.5% to 71.7% [1,3]. Similarly, previous studies have reported comparable rates of in-hospital mortality in this patient population [1,3,6]. These findings highlight the importance of identifying parameters capable of predicting both emergency department and in-hospital mortality in patients with EDCA.

According to the revised European Working Group on Sarcopenia in Older People (EWGSOP2) consensus, sarcopenia is a progressive and generalized skeletal muscle disorder in which low muscle strength is the primary parameter, while low muscle quantity or quality confirms the diagnosis and impaired physical performance indicates severe sarcopenia [7]. It is associated with several adverse health outcomes and poor clinical prognosis [8]. Various approaches have been investigated to assess muscle quantity and related components of sarcopenia; however, some of these methods are limited by cost, accessibility, or difficulties in routine clinical application [9]. Imaging-based measurements of skeletal muscle may provide information regarding muscle quantity, but they cannot independently establish a diagnosis of sarcopenia. In recent years, temporalis muscle thickness (TMT), which can easily be measured on cranial computed tomography images, has been investigated as an imaging-based morphometric surrogate associated with reduced muscle mass and frailty [10]. A limited number of studies have examined the association between TMT and in-hospital or 30-day survival in patients with cardiac arrest; however, these studies have not demonstrated a consistent significant association [11,12]. Furthermore, the relationship between TMT and emergency department and in-hospital mortality specifically in patients with EDCA remains unclear. Evaluating this association may provide further insight into the potential prognostic role of TMT in this distinct cardiac arrest population.

The primary aim of this study was to evaluate the association between TMT and emergency department mortality among patients who experienced cardiac arrest in the emergency department and had an eligible cranial CT examination during the same emergency department encounter. The secondary aim was to investigate its association with in-hospital mortality in this patient group.

## 2. Materials and Methods

This study was conducted on patients presenting to the emergency department of a tertiary care hospital. The study was designed as a retrospective observational cohort study. This study was conducted according to the guidelines of the Declaration of Helsinki and was approved by the Research Ethics Committee of the Faculty of Medicine at Hitit University on 3 June 2025 (decision number = 2025-127). Informed consent was waived due to the retrospective design of the study. Patients who presented to the emergency department and experienced cardiac arrest between 1 June 2022 and 31 May 2025 were included in the study. Patients who did not undergo non-contrast brain computed tomography (CT) at any time during the emergency department visit (either before or after cardiac arrest), those who suffered cardiac arrest following trauma, patients younger than 18 years of age, patients with incomplete data that precluded assessment of the primary study variables or outcomes, and those whose brain CT images were not suitable for evaluation were excluded from the study. Achievement of ROSC was not required for inclusion; patients meeting the eligibility criteria were included regardless of whether ROSC was achieved. Subsequently, patients were categorized as EDCA and OHCA. EDCA was defined as cardiac arrest occurring after arrival to the emergency department, whereas OHCA was defined as cardiac arrest occurring before emergency department arrival. After application of the eligibility criteria, the remaining patients were categorized according to arrest location, and only patients with EDCA were included in the final analysis.

**Figure 1:**
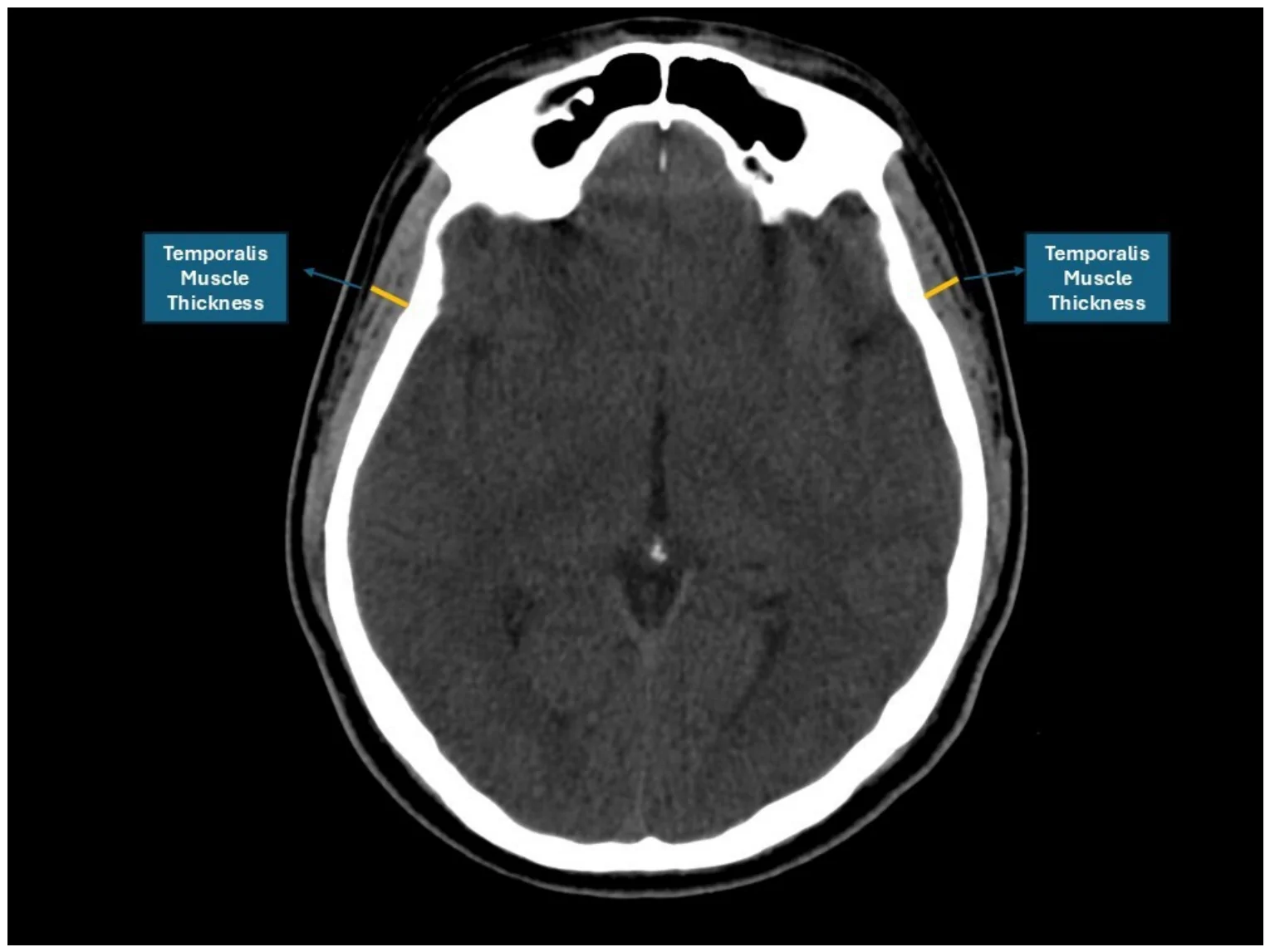
Representative axial non-contrast brain CT image demonstrating bilateral temporalis muscle thickness measurement. Measurements were obtained manually at a level approximately 0.5 cm above the superior orbital rim adjacent to the Sylvian fissure, using a window setting of 20–200 Hounsfield units.

Emergency department data of the patients were reviewed through the hospital information management system (HIMS) by a single researcher, and TMT was calculated by measuring the non-contrast brain CT images of patients who met the inclusion criteria at emergency department admission. Non-contrast brain CT images obtained at any time during the emergency department visit (either before or after cardiac arrest) were used for the measurements. Brain CT was not performed according to a standardized study protocol; rather, the decision to obtain CT was based on the treating physician’s clinical judgment and the patient’s clinical presentation. Eligible CT examinations included scans obtained during the same emergency department encounter either before or after the cardiac arrest event. Accordingly, the timing of CT relative to cardiac arrest was retrospectively classified as pre-arrest or post-arrest. The CT device used in the emergency department was a General Electric (GE) Healthcare Optima CT660 (Waukesha, WI, USA) with 128 slices, with the following acquisition parameters: helical thickness, 1.25 mm; interval, 1.25 mm; tube voltage, 120 kV; tube current, 50–400 mA (automatic mA); pitch, 0.969:1; rotation time, 0.5 s; collimation, 20 mm; matrix size, 512 × 512; and field of view (FOV), 32 cm. Measurements were performed manually and bilaterally on the CT slice located 0.5 cm above the superior orbital rim adjacent to the Sylvian fissure, using a window setting of 20–200 Hounsfield units (Figure 1).

Measurements were conducted independently for each image by an emergency medicine specialist and a radiology specialist, both of whom had at least five years of clinical experience and were experienced in temporalis muscle measurement. Both readers performed the TMT measurements independently and were blinded to the patients’ clinical outcomes and to each other’s measurements. All measurements were recorded. Interobserver agreement between the emergency medicine specialist and the radiology specialist was subsequently evaluated to assess whether measurements obtained by the emergency medicine specialist could be used interchangeably with those of the radiology specialist. In the study, the mean value of the right and left TMT measured by the emergency medicine specialist was used for analysis. The emergency medicine specialist’s measurements were selected for the primary analyses to reflect the prognostic performance of TMT measured by a single emergency physician, whereas the radiologist’s measurements were used independently to assess interobserver reliability rather than to generate an averaged consensus measurement.

Patient data including age, sex, comorbidities, presenting arrest rhythms (shockable or non-shockable), presumed cause of cardiac arrest, duration of cardiopulmonary resuscitation (CPR) (minutes), and laboratory parameters obtained at emergency department admission (white blood cell count, neutrophil count, lymphocyte count, hemoglobin, hematocrit, platelet count, albumin, blood urea nitrogen, creatinine, glomerular filtration rate, aspartate transaminase, alanine transaminase, sodium, potassium, calcium, C-reactive protein, troponin-I, international normalized ratio, ionized calcium, venous pH, venous partial pressure of carbon dioxide, venous bicarbonate, venous lactate, and venous base excess), as well as mortality outcomes (emergency department mortality and in-hospital mortality), were independently extracted and recorded from the HIMS by two researchers who were blinded to the CT imaging results. CPR duration was defined as the time from initiation of cardiopulmonary resuscitation until either achievement of ROSC or termination of resuscitative efforts in patients in whom ROSC was not achieved. This approach ensured blinding within the study. Emergency department mortality was defined as death occurring in the emergency department before transfer to an intensive care unit, another ward, or another inpatient clinical unit. In-hospital mortality was defined as death occurring at any time from emergency department admission until hospital discharge, including deaths occurring in the emergency department. Accordingly, emergency department mortality represented a subset of overall in-hospital mortality, and the two outcomes were therefore nested rather than mutually exclusive. Subsequently, the association between TMT and mortality outcomes was statistically analyzed.

All statistical evaluations were conducted using Jamovi software (version 2.7.15). The distributional properties of continuous variables were examined through the Shapiro–Wilk normality test in conjunction with graphical assessment using histograms. Continuous data were expressed as median (Q1–Q3), where Q1 and Q3 represent the 25th and 75th percentiles, respectively, whereas categorical variables were reported as counts and percentages. Missing data were not imputed. Descriptive analyses were performed using the available data for each variable. Multivariable logistic regression analyses were performed using complete cases for the variables included in each model. Because age, sex, CPR duration, TMT, and mortality outcomes were available for all 129 patients, the full cohort was included in both multivariable models. As TMT values did not demonstrate a normal distribution, group comparisons were carried out using the Mann–Whitney U test. The discriminatory performance of TMT in predicting both emergency department mortality and in-hospital mortality was analyzed by constructing receiver operating characteristic (ROC) curves. Cut-off thresholds were identified based on the maximum Youden index. For each selected threshold, classification performance indicators—including sensitivity, specificity, positive predictive value (PPV), negative predictive value (NPV), positive likelihood ratio (+LR), and negative likelihood ratio (−LR)—were calculated. Interobserver reliability between measurements obtained by the emergency medicine specialist and the radiology specialist was assessed using a two-way mixed effects intraclass correlation coefficient (ICC) for absolute agreement based on single measurements. Univariable binary logistic regression analyses were initially performed to examine the association between candidate variables and mortality outcomes. For the revised multivariable analyses, covariate selection was based on a parsimonious, clinically driven approach rather than solely on univariable statistical significance. TMT was retained as the primary predictor of interest. Age and sex were included a priori as potential confounders because both may influence temporalis muscle dimensions, while CPR duration was included because of its established prognostic relevance following cardiac arrest. Given the limited number of emergency department deaths (*n* = 30), the primary multivariable model was deliberately restricted to these four predictors to reduce the risk of overfitting. Presenting rhythm was also considered as a potential prognostic covariate; however, only 14 patients (10.9%) had a shockable rhythm. Given this sparse distribution and the limited number of emergency department mortality events, it was not included in the primary multivariable model to avoid further model instability and overfitting. Multicollinearity was assessed using variance inflation factors (VIFs), and no relevant multicollinearity was identified. The linearity-in-the-logit assumption was evaluated for continuous predictors. Model calibration was assessed using the Hosmer–Lemeshow goodness-of-fit test, and model discrimination was evaluated using the area under the receiver operating characteristic curve (AUC). Model fit was additionally summarized using the likelihood-ratio chi-square test and Nagelkerke R^2^. Statistical significance was defined as a two-tailed *p* value of less than 0.05.

## 3. Results

**Figure 2:**
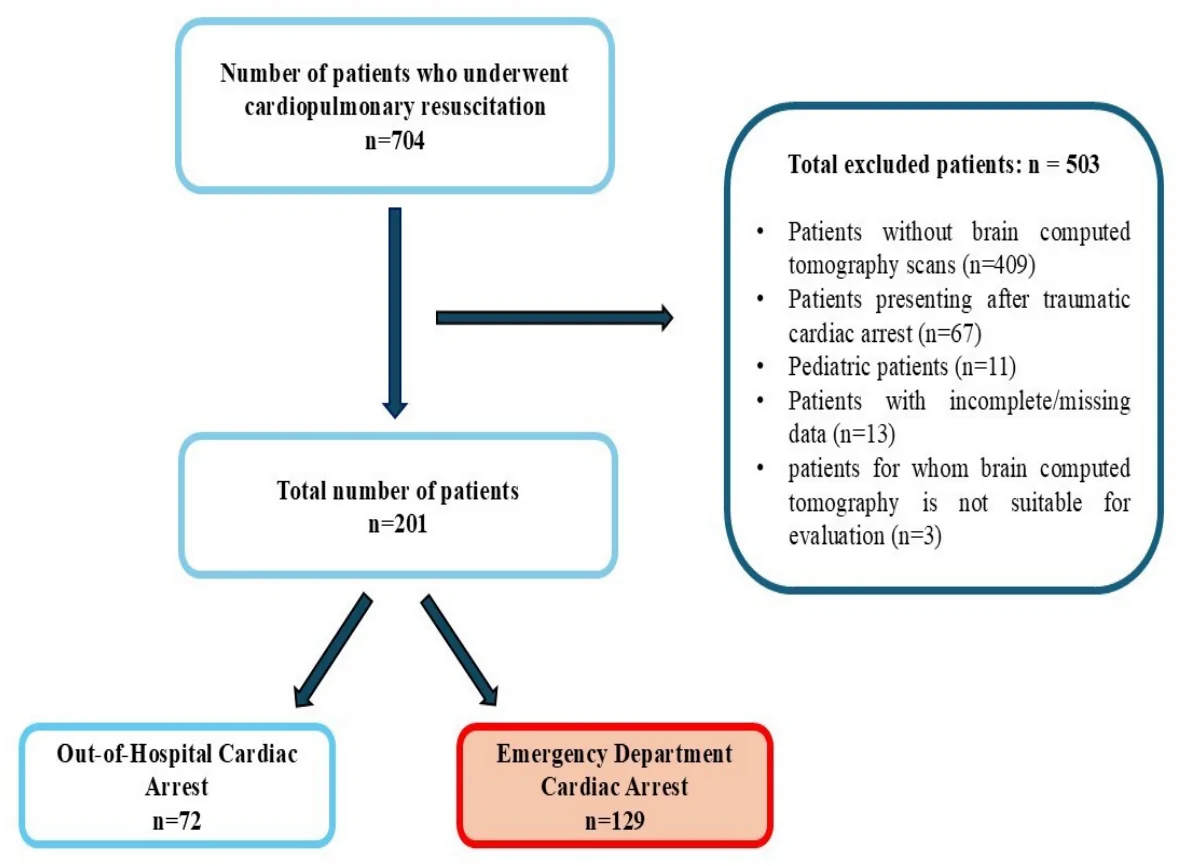
Flowchart of the study design.

During the study period, a total of 704 patients who experienced cardiac arrest were evaluated. Of these, 503 patients were excluded: 409 because an eligible cranial CT was unavailable, 67 because of traumatic cardiac arrest, 11 because they were younger than 18 years of age, 13 because of incomplete or missing data, and 3 because the cranial CT images were unsuitable for TMT measurement. Among the remaining 201 patients who suffered cardiac arrest, 72 were classified as OHCA. Ultimately, 129 patients who suffered EDCA were included in the final analysis (Figure 2). Among the 129 included patients, 10 (7.8%) had at least one missing laboratory value. Missingness for individual laboratory variables ranged from 0.8% to 3.1%. No data were missing for age, sex, CPR duration, TMT measurements, emergency department mortality, or in-hospital mortality.

Among the 129 included patients, cranial CT was obtained before the cardiac arrest event in [*n* = 55, 43%] and after the cardiac arrest event in [*n* = 74, 57%]. All post-arrest CT examinations were obtained after the return of spontaneous circulation. Of the patients included in the study, 47.3% (*n* = 61) were female, and the median age was 74 years (Q1–Q3: 65–83). The most frequently observed comorbidities were hypertension (*n* = 106, 82.2%), coronary artery disease (*n* = 74, 57.4%), and diabetes mellitus (*n* = 62, 48.1%). Cardiac arrest with a shockable rhythm was observed in 10.9% (n = 14) of the patients, and the median duration of CPR was 20 min (Q1–Q3: 10–30). Overall, emergency department mortality occurred in 30 patients (23.3%), while in-hospital mortality was observed in 106 patients (82.2%). The median value of the mean right and left TMT measured by the emergency medicine specialist was 4.22 mm (Q1–Q3: 3.31–5.44), whereas the median TMT measured by the radiology specialist was 4.10 mm (Q1–Q3: 3.20–5.20). Detailed patient characteristics are presented in Table 1. Very strong agreement was observed between TMT measurements performed by the emergency medicine specialist and the radiology specialist (ICC = 0.914 [95% CI 0.875–0.940], *p* < 0.001) [13].

When patients were evaluated according to emergency department mortality status, non-survivors were older than survivors (median 77.5 years [Q1–Q3: 72.25–86.75] vs. 73 years [Q1–Q3: 61–82], *p* = 0.013) and had a significantly longer CPR duration (median 45 min [Q1–Q3: 45–45] vs. 15 min [Q1–Q3: 6–25], *p* < 0.001). Female sex (60.0% vs. 43.4%, *p* = 0.111) and shockable presenting rhythm (10.0% vs. 11.1%, *p* = 1.000) did not differ significantly between non-survivors and survivors. Pre-arrest cranial CT was more frequent among emergency department non-survivors than survivors (70.0% vs. 34.3%), whereas post-arrest CT was more frequent among survivors (30.0% vs. 65.7%; *p* < 0.001). Detailed results are presented in Table 2.

In our study, the median TMT was 3.29 mm (Q1–Q3: 2.63–3.95) in patients who died in the emergency department, compared with 4.69 mm (Q1–Q3: 3.70–5.65) in survivors. TMT was found to be significantly lower in patients who died in the emergency department (*p* < 0.001). When in-hospital mortality was evaluated, the median TMT was 4.06 mm (Q1–Q3: 3.26–5.34) in non-survivors and 5.00 mm (Q1–Q3: 4.21–5.85) in survivors. Similarly, TMT was significantly lower in patients who died in the hospital (*p* = 0.040). Detailed results are presented in Table 3.

**Figure 3:**
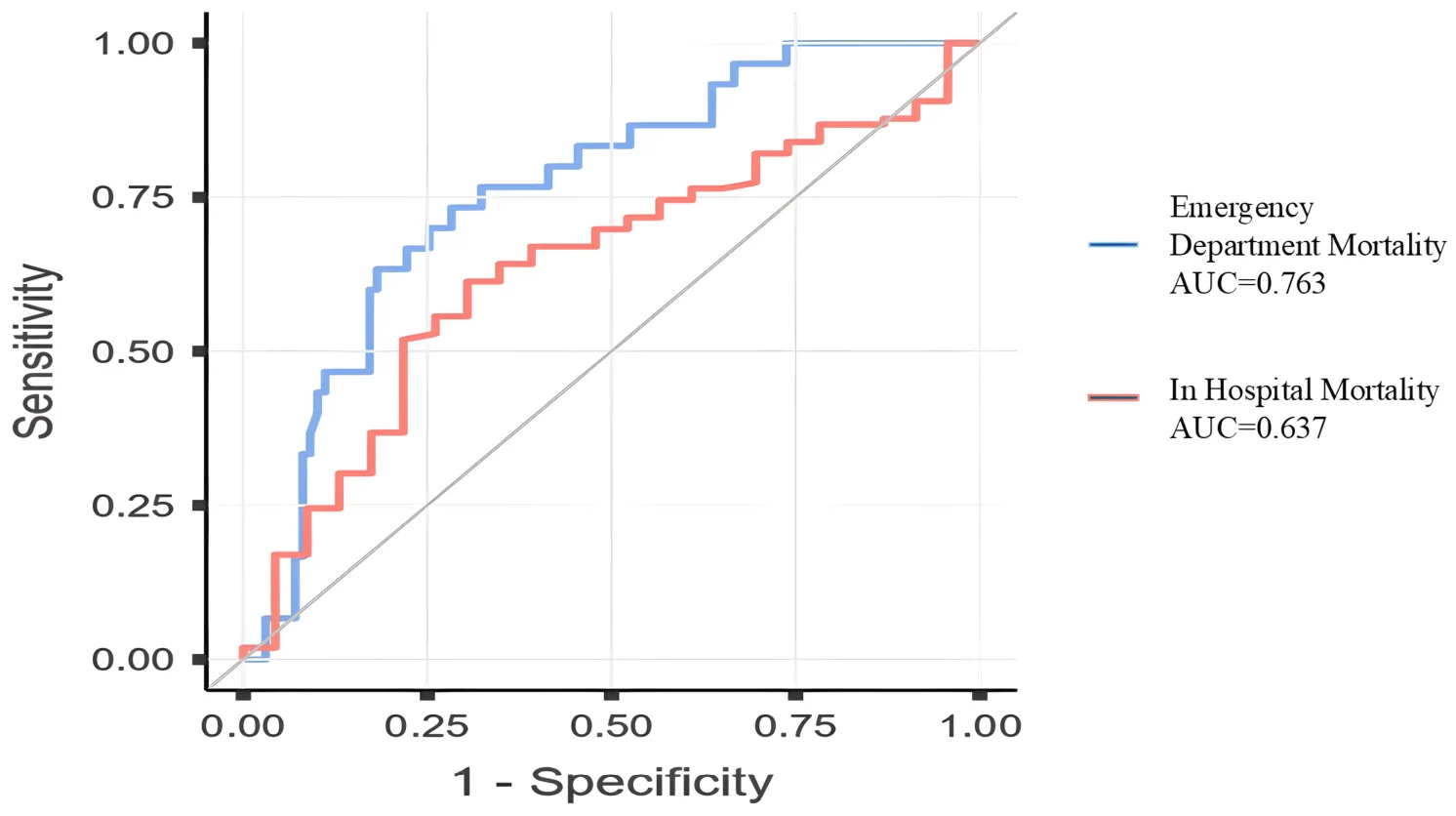
Receiver operating characteristic curves of temporalis muscle thickness in predicting emergency department and in-hospital mortality.

The predictive performance of TMT for emergency department mortality and in-hospital mortality was evaluated using ROC curve analysis. The AUC for predicting emergency department mortality was 0.763 (95% CI 0.671–0.856), while the AUC for predicting in-hospital mortality was 0.637 (95% CI 0.516–0.757) (Figure 3).

According to the Youden index, the optimal cut-off value for predicting emergency department mortality was approximately 3.4 mm. At this threshold, sensitivity was 63.33%, specificity was 81.82%, PPV was 51.35%, NPV was 88.04%, +LR was 3.483, and −LR was 0.448. The corresponding exploratory threshold for in-hospital mortality was approximately 4.5 mm, with a sensitivity of 61.32%, a specificity of 69.57%, a PPV of 90.28%, an NPV of 28.07%, a +LR of 2.015, and a −LR of 0.556. These ROC-derived thresholds were derived and evaluated in the same cohort and should therefore be considered exploratory and require external validation before any clinical application. Detailed results are presented in Table 4.

Multivariable logistic regression analysis was performed using the prespecified final model including age, sex, CPR duration, and TMT. For emergency department mortality, 30 deaths occurred among 129 patients. In the final model, CPR duration (adjusted OR per 1 min increase, 1.31; 95% CI, 1.15–1.48; *p* < 0.001) and TMT (adjusted OR per 1 mm increase, 0.34; 95% CI, 0.133–0.886; *p* = 0.027) were independently associated with mortality, whereas age (adjusted OR per 1-year increase, 0.98; 95% CI, 0.90–1.07; *p* = 0.633) and female sex (adjusted OR, 3.30; 95% CI, 0.42–26.2; *p* = 0.259) were not (χ^2^ = 107.57, *p* < 0.001). Given the limited number of emergency department mortality events, these multivariable findings should be considered exploratory.

For in-hospital mortality, which occurred in 106 of 129 patients, age (adjusted OR per 1-year increase, 1.05, 95% CI 1.01–1.09, *p* = 0.010) and CPR duration (adjusted OR per 1 min increase, 1.07, 95% CI 1.02–1.12, *p* = 0.014) were independently associated with mortality, whereas TMT was not (adjusted OR per 1 mm increase, 1.07, 95% CI 0.78–1.47; *p* = 0.681) (χ^2^ = 37.6, *p*  <  0.001). The results of the logistic regression analyses are presented in Table 5.

## 4. Discussion

In this CT-selected cohort of patients with EDCA who had eligible cranial CT imaging during the same emergency department encounter, TMT was significantly lower among patients who died in the emergency department and among those who died in-hospital. While TMT remained independently associated with emergency department mortality after adjustment for age, sex, and CPR duration, no independent association was observed with in-hospital mortality. Sarcopenia is a progressive and generalized skeletal muscle disorder characterized primarily by reduced muscle strength, with reduced muscle quantity or quality used to confirm the diagnosis according to the EWGSOP2 consensus [7]. Sarcopenia has been evaluated in patients using various methods, including measurements of different muscle groups [9]. Several studies have investigated the relationship between reduced muscle mass and prognosis in patients with cardiac arrest. One study demonstrated that decreased thoracic muscle volume was associated with poor neurological outcomes in patients with out-of-hospital cardiac arrest, while another study showed that skeletal muscle loss in patients who suffered cardiac arrest during hospitalization was similarly associated with poor long-term neurological outcomes [14,15].

TMT has been investigated as an imaging-based morphometric surrogate associated with muscle mass and frailty, rather than as a standalone diagnostic measure of sarcopenia [10]. In a study conducted by Tam et al., it was reported that TMT measured on non-contrast brain computed tomography in patients with cardiac arrest showed a significant correlation with the pre-arrest clinical frailty score [11]. The association between frailty and poor outcomes following cardiac arrest has been previously demonstrated. However, the study by Tam et al. specifically highlights the correlation between frailty and TMT in patients with cardiac arrest. Considering that the use of frailty indices in the emergency department is often not feasible, this relationship underscores the importance of TMT measurement as a practical tool for assessing frailty in emergency department settings [11,16,17].

On the other hand, Tam et al. did not identify an association between TMT and survival to hospital discharge or favorable functional outcomes. Similarly, Hongo et al. examined the relationship between TMT and 30-day survival in patients admitted to the intensive care unit following cardiac arrest and found no significant association between TMT and 30-day survival [11,12]. In our study, we investigated the association between TMT and both emergency department and in-hospital mortality among patients with EDCA who had eligible cranial CT imaging during the same emergency department encounter. To the best of our knowledge, this study represents the first investigation specifically evaluating the association between TMT and mortality in patients with EDCA. Previous studies evaluated broader resuscitated cardiac arrest populations or survivors of OHCA, whereas the present study focused specifically on patients who suffered cardiac arrest in the emergency department and evaluated both emergency department and in-hospital mortality. Contrary to the findings of Tam et al. and Hongo et al., our results demonstrated that decreased TMT was significantly associated with both emergency department and in-hospital mortality [11]. This discrepancy may be attributable to the inclusion of more heterogeneous patient populations and a focus on longer-term outcomes in previous studies. In contrast, the selection of a more homogeneous and isolated patient group in our study, i.e., EDCA patients, may have contributed to a clearer demonstration of the prognostic significance of TMT.

Furthermore, TMT remained independently associated with emergency department mortality after multivariable adjustment, whereas no independent association was observed with in-hospital mortality. The stronger observed association of TMT with emergency department mortality than with cumulative in-hospital mortality should be interpreted cautiously, because emergency department deaths were included within the in-hospital mortality outcome rather than representing an independent comparison group. Lower TMT may reflect reduced muscle reserve, nutritional vulnerability, or broader baseline frailty, which could influence tolerance to the profound physiological and metabolic stress associated with cardiac arrest and resuscitation. Among patients surviving the emergency department phase, subsequent mortality during hospitalization may additionally be influenced by neurological injury, infection, multiorgan dysfunction, intensive care complications, and treatment-related processes [18,19]. However, post-transfer mortality was not analyzed as a separate outcome in the present study; therefore, these considerations remain hypothesis-generating [19]. Because TMT is influenced by demographic and constitutional factors, particularly age and sex, these variables were included a priori in the revised multivariable model. Importantly, the association between lower TMT and emergency department mortality persisted after adjustment for age, sex, and CPR duration, suggesting that the observed relationship was not explained solely by differences in age or sex. Nevertheless, TMT may partly reflect broader biological vulnerability, including reduced muscle mass, nutritional status, body composition, and frailty. Therefore, the present findings should not be interpreted as establishing a direct causal relationship between TMT and mortality, but rather as demonstrating an exploratory association after adjustment for the measured covariates. The relatively high Nagelkerke R^2^ observed for the emergency department mortality model warrants cautious interpretation. CPR duration was strongly associated with emergency department mortality; however, this variable should not be considered a purely baseline prognostic factor. In patients in whom ROSC was not achieved, CPR duration extended until termination of resuscitative efforts and was therefore partly determined by the clinical outcome itself. This relationship may have contributed to the high apparent explanatory performance of the model. Accordingly, the model performance should not be interpreted as evidence of broad prognostic generalizability. Finally, although TMT was associated with emergency department mortality in the present cohort, we believe that it is not appropriate to use TMT alone to determine mortality or to guide decisions regarding the continuation or termination of resuscitation efforts. The present findings demonstrate an association between TMT and emergency department mortality after adjustment for the measured covariates; however, they do not establish that TMT provides incremental prognostic information beyond established clinical and resuscitation variables. Therefore, the clinical utility of TMT and its potential contribution to multivariable risk-stratification models require evaluation in larger, independently validated cohorts.

Our study has several limitations. First, due to its retrospective and single-center design, the data were derived from a relatively limited population. This may restrict the generalizability of the findings to the broader population. Another limitation is that detailed time intervals from cardiac arrest or ROSC to emergency department disposition or death, as well as length of hospital stay, were not available in the retrospective dataset; therefore, temporal differences in mortality trajectories could not be further characterized. Additionally, although presenting rhythm, CPR duration, and presumed arrest etiology were available, several established cardiac arrest-related prognostic factors, including witnessed status, time to CPR initiation, no-flow/low-flow durations, and pre-arrest functional status, and detailed ROSC characteristics, including sustained ROSC status and duration, could not be reliably evaluated from the retrospective records. Presumed arrest etiology was recorded descriptively; however, the large number of heterogeneous categories and small numbers within individual categories precluded its reliable inclusion in the parsimonious multivariable model. Therefore, residual confounding by unmeasured or incompletely characterized arrest-related factors cannot be excluded. An important limitation of this study is the CT-based selection of the study population. A substantial proportion of screened patients did not undergo cranial CT and were therefore excluded. Because CT acquisition was clinically determined rather than protocolized, patients who underwent cranial CT may have differed systematically from those who did not with respect to their initial presentation, suspected arrest etiology, neurological status, resuscitation characteristics, clinical stability, and prognosis. In particular, the exclusion of 409 patients without eligible cranial CT may have introduced substantial selection bias. Furthermore, because post-arrest CT required sufficient clinical stability to undergo diagnostic imaging, survivorship bias cannot be excluded. Therefore, the findings of the present study should not be generalized to the overall EDCA population but should primarily be interpreted in the context of EDCA patients for whom eligible cranial CT imaging was clinically available during the emergency department encounter. Another limitation is that the indication for cranial CT could not be reliably categorized for all patients because this information was inconsistently documented in retrospective medical records. Furthermore, temporalis muscle measurements were performed using cranial CT images, which represents another limitation. In a study conducted by Yılmaz et al., only a moderate correlation was observed between temporalis muscle measurements obtained by CT and ultrasonography, suggesting that ultrasonography may represent a useful alternative method for TMT assessment [20]. This discrepancy in measurement modalities may have influenced the relationship between TMT and clinical outcomes in our study. Nevertheless, when cranial CT is obtained for clinical indications, TMT can be retrospectively derived from already available imaging without requiring additional radiation exposure or dedicated imaging. Another limitation is the limited number of emergency department mortality events. Despite restricting the primary multivariable model to four clinically selected predictors, only 30 emergency department deaths occurred, corresponding to approximately 7.5 outcome events per predictor. Therefore, overfitting and instability of the estimated regression coefficients cannot be excluded, as also reflected by the relatively wide confidence intervals for some estimates. No formal bootstrap internal validation or penalized regression was performed; therefore, optimism in the estimated model performance cannot be excluded. The multivariable findings should therefore be regarded as exploratory associations within the measured covariates and require confirmation and internal and external validation in larger independent cohorts. In addition, body mass index, direct measures of nutritional status, muscle strength, body composition, pre-arrest functional status, and validated frailty measures were not available because of the retrospective study design. As these factors may influence both TMT and clinical outcomes, residual confounding by these factors cannot be excluded. Furthermore, TMT represents a single imaging-based morphometric measurement and does not constitute a diagnosis of sarcopenia, as muscle strength, physical performance, and other components required for formal sarcopenia assessment were not evaluated in this retrospective study. In addition, the ROC-derived thresholds were selected and evaluated within the same relatively small cohort without internal or external validation. Therefore, the approximately 3.4 mm and 4.5 mm ROC-derived thresholds should not be interpreted as established clinical cut-offs or as stand-alone clinical decision thresholds, but rather as exploratory values requiring external validation in independent cohorts. Finally, no formal external validation was performed; therefore, the multivariable findings and ROC-derived thresholds should be considered exploratory and require confirmation in independent cohorts. Importantly, to the best of our knowledge, this study represents the first investigation to evaluate the association between TMT and prognosis in patients with EDCA. Therefore, it may be considered a preliminary study that could provide a foundation for future research. Future studies may evaluate the feasibility and prognostic value of temporalis muscle measurements using point-of-care ultrasonography after ROSC or once the patient is clinically stabilized, rather than during active resuscitation.

## 5. Conclusions

In conclusion, among patients with EDCA who had eligible cranial CT imaging during the same emergency department encounter, lower TMT was independently associated with emergency department mortality after adjustment for the measured covariates, whereas no independent association was observed with cumulative in-hospital mortality. Given the limited number of outcome events and the absence of internal or external validation, these findings should be considered exploratory and require confirmation in larger independent cohorts. Future studies should focus on larger patient populations and evaluate ultrasonographic TMT measurement after ROSC or during subsequent clinical stabilization, while further assessing its prognostic value and validating the exploratory ROC-derived thresholds in independent cohorts. Such approaches may allow for a more detailed assessment of the extent to which TMT is associated with mortality.

## Figures and Tables

**Table 1:** Baseline characteristics and descriptive statistics of the study population.

	Total Patients (n = 129)
**Sex, female, n (%)**	61 (47.3)
**Age, years, median (Q1–Q3)**	74 (65–83)
**Comorbidities, n (%)**	
Hypertension	106 (82.2)
Coronary artery disease	74 (57.4)
Congestive heart failure	37 (28.7)
Arrhythmia	30 (23.3)
Hyperlipidemia	45 (34.9)
Diabetes mellitus	62 (48.1)
Chronic obstructive pulmonary disease	33 (25.6)
Cerebrovascular disease	37 (28.7)
Chronic renal disease	19 (14.7)
Chronic liver disease	6 (4.7)
Malignancy	18 (14.0)
**Rhythm, shockable, n (%)**	14 (10.9)
**Cardiopulmonary resuscitation duration, min, median (Q1–Q3)**	20 (10–30)
**Emergency department mortality, n (%)**	30 (23.3)
**In-hospital mortality, n (%)**	106 (82.2)
**Laboratory findings, median (Q1–Q3)**	
White blood cell, 10^3^/µL	12.9 (9.32–16.68)
Neutrophil, 10^3^/µL	8.91 (4.96–12.60)
Lymphocyte, 10^3^/µL	1.81 (0.70–4.55)
Hemoglobin, g/dL	11.6 (10.3–14.1)
Hematocrit, %	37.7 (33.5–43.8)
Platelet, 10^3^/µL	205 (142–273)
Albumin, g/L	33.5 (26.75–38.25)
Blood urea nitrogen, mg/dL	35.5 (20.75–61.00)
Creatinine, mg/dL	1.50 (1.00–2.25)
Glomerular filtration rate, mL/min/1.73 m^2^	38.5 (23.00–67.25)
AST, U/L	43.5 (24.00–99.75)
ALT, U/L	25.0 (13.00–63.25)
Sodium, mmol/L	138 (134.75–141.25)
Potassium, mmol/L	4.79 (4.07–5.65)
Calcium, mg/dL	8.80 (8.18–9.30)
C-reactive protein, mg/dL	37.7 (6.60–133.00)
Troponin I, ng/L	223 (112–628.5)
INR	1.17 (1.06–1.47)
Ionized calcium, mmol/L	1.06 (0.99–1.12)
Venous pH	7.22 (7.08–7.47)
Venous PCO_2_, mmHg	44.0 (35.30–61.13)
Venous HCO_3_^−^, mEq/L	16.4 (11.58–20.03)
Venous lactate, mmol/L	6.93 (3.94–10.18)
Venous base excess, mmol/L	−9.25 (−17.60–−5.10)
**Timing of cranial CT imaging, n (%)**	
Before cardiac arrest	55 (43)
After cardiac arrest	74 (57)
**Presumed cause of cardiac arrest, n (%)**	
Pneumonia-associated sepsis	24 (18.6)
Respiratory failure	22 (17.1)
Sepsis	14 (10.9)
Pulmonary edema	12 (9.3)
Myocardial infarction	11 (8.5)
Pulmonary embolism	7 (5.4)
Cerebrovascular event	6 (4.7)
Acute kidney injury	5 (3.9)
Aspiration pneumonia	5 (3.9)
Anemia	3 (2.3)
Hyperkalemia	3 (2.3)
Hypernatremia	2 (1.6)
Ventricular fibrillation	2 (1.6)
Acute arterial occlusion	1 (0.8)
Ruptured aortic aneurysm	1 (0.8)
Decompensated heart failure	1 (0.8)
Diabetic ketoacidosis	1 (0.8)
Epilepsy	1 (0.8)
Gastrointestinal bleeding	1 (0.8)
Hypokalemia	1 (0.8)
Mesenteric ischemia	1 (0.8)
Multiorgan failure	1 (0.8)
Cirrhosis	1 (0.8)
Ileus	1 (0.8)
Intoxication	1 (0.8)
Intracranial hemorrhage	1 (0.8)
**Temporalis muscle thickness, median (Q1–Q3)**	
**Radiologist-performed measurements**	
Right side, mm	4.40 (3.30–5.30)
Left side, mm	3.80 (3.00–5.10)
Mean, mm	4.10 (3.20–5.20)
**EM specialist-performed measurements**	
Right side, mm	4.34 (3.36–5.48)
Left side, mm	4.18 (3.22–5.46)
Mean, mm	4.22 (3.31–5.44)

AST = aspartate transaminase; ALT = alanine transaminase; INR = international normalized ratio; PCO_2_ = partial carbon dioxide; HCO_3_^−^ = bicarbonate; n = number; Q1 = first quartile; Q3 = third quartile. Note: Text written in bold represents the main headings in the category. Available-case denominators were *n* = 125 for INR and ionized calcium; *n* = 127 for troponin-I, CRP, and lactate; and *n* = 128 for BUN, creatinine, GFR, AST, ALT, albumin, sodium, potassium, calcium, base excess, HCO_3_, PCO_2_, and pH. All other reported variables had complete data (*n* = 129).

**Table 2:** Key clinical characteristics according to emergency-department-mortality status.

	ED Survivor (n = 99)	ED Non-Survivor (n = 30)	*p* Value
Age, years, median (Q1–Q3)	73 (61–82)	77.5 (72.25–86.75)	0.013
Female sex, n (%)	43 (43.4%)	18 (60.0%)	0.111
Shockable rhythm, n (%)	11 (11.1%)	3 (10.0%)	1.000
CPR duration, min, median (Q1–Q3)	15 (6–25)	45 (45–45)	<0.001
CT before cardiac arrest, n (%)	34 (34.3%)	21 (70.0%)	<0.001
CT after cardiac arrest, n (%)	65 (65.7%)	9 (30.0%)	<0.001

ED: emergency department; n = number; Q1 = first quartile; Q3 = third quartile; CPR: cardiopulmonary resuscitation; CT: computed tomography.

**Table 3:** Temporalis muscle thickness according to emergency-department- and in-hospital-mortality status.

	Emergency Department Mortality			
	**Total Patients (n = 129)**	**Survivors (n = 99)**	**Non-Survivors (n = 30)**	*p* Value *
Temporalis muscle thickness, mm, median (Q1–Q3)	4.22 (3.31–5.44)	4.69 (3.70–5.65)	3.29 (2.63–3.95)	<0.001
	**In-Hospital Mortality**			
	**Total Patients (n = 129)**	**Survivors (n = 23)**	**Non-Survivors (n = 106)**	
Temporalis muscle thickness, mm, median (Q1–Q3)	4.22 (3.31–5.44)	5.00 (4.21–5.85)	4.06 (3.26–5.34)	0.040

n = number; Q1 = first quartile; Q3 = third quartile; mm = millimeters. *p* < 0.05 was considered statistically significant. * According to the Mann–Whitney U test results.

**Table 4:** Discriminative performance of temporalis muscle thickness for emergency department and in-hospital mortality.

	Cut-Off	AUC (95% CI)	SEN, % (95% CI)	SPE, % (95% CI)	PPV, % (95% CI)	NPV, % (95% CI)	+LR (95% CI)	−LR (95% CI)
Emergency department mortality								
Temporalis muscle thickness, mm	≤3.4	0.763 (0.671–0.856)	63.33 (43.86–80.07)	81.82 (72.80–88.85)	51.35 (39.06–63.48)	88.04 (82.01–92.24)	3.483 (2.115–5.736)	0.448 (0.277–0.724)
In-hospital mortality								
Temporalis Muscle Thickness, mm	≤4.5	0.637 (0.516–0.757)	61.32 (51.37–70.62)	69.57 (47.08–86.79)	90.28 (83.10–94.61)	28.07 (21.38–35.90)	2.015 (1.067–3.806)	0.556 (0.387–0.798)

AUC = area under curve; SEN = sensitivity; SPE = specificity; PPV = positive predictive value; NPV = negative predictive value; +LR = positive likelihood ratio; −LR = negative likelihood ratio; CI = confidence interval; mm = millimeters.

**Table 5:** Univariable and final multivariable logistic regression analyses of factors associated with emergency department and in-hospital mortality.

	Univariable Analysis		Multivariable Analysis	
	Adjusted OR (95% CI)	*p* Value	Adjusted OR (95% CI)	*p* Value
Emergency department mortality				
Age, per year	1.05 (1.01–1.09)	0.008	0.98 (0.90–1.07)	0.633
Female sex	1.95 (0.85–4.49)	0.114	3.30 (0.42–26.2)	0.259
CPR duration, per min	1.25 (1.15–1.35)	<0.001	1.307 (1.15–1.48)	<0.001
Temporalis muscle thickness, per mm	0.477 (0.326–0.698)	<0.001	0.343 (0.133–0.886)	0.027
In-hospital mortality				
Age, per year	1.053 (1.02–1.09)	0.002	1.05 (1.01–1.09)	0.010
Female sex	1.875 (0.73–4.79)	0.189	2.06 (0.70–6.10)	0.191
CPR duration, per min	1.06 (1.02–1.10)	0.005	1.07 (1.02–1.12)	0.014
Temporalis muscle thickness, per mm	0.797 (0.614–1.03)	0.087	1.07 (0.78–1.47)	0.681

Emergency department mortality: 30 events/129 patients; likelihood-ratio χ^2^ = 107.57, *p*  <  0.001, Nagelkerke R^2^  =  0.854. In-hospital mortality; 106 events/129 patients; likelihood-ratio χ^2^ = 37.6, *p*  <  0.001, Nagelkerke R^2^  =  0.252. The final multivariable model included age, sex, CPR duration, and TMT, selected a priori based on clinical relevance and potential confounding. ORs for age, CPR duration, and TMT are expressed per 1-year, 1 min, and 1 mm increase, respectively. Given the limited number of emergency department deaths, the multivariable analysis should be considered exploratory. OR = odds ratio; CI = confidence interval.

## Data Availability

The data that support the findings of this study are available from the corresponding author upon reasonable request. The data are not publicly available due to patient privacy and confidentiality considerations.

## Abbreviations

The following abbreviations are used in this manuscript:

IHCA	In-hospital cardiac arrest
OHCA	Out-of-hospital cardiac arrest
EDCA	Emergency department cardiac arrest
TMT	Temporalis muscle thickness
CT	Computed tomography
HIMS	Hospital information management system
GE	General Electric
FOV	Field of view
IQR	Interquartile ranges
ROC	Receiver operating characteristics
PPV	Positive predictive value
NPV	Negative predictive value
+LR	Positive likelihood ratio
−LR	Negative likelihood ratio
ICC	Intraclass correlation coefficient
N	Number
CPR	Cardiopulmonary resuscitation
AUC	Area under curve
